# Nicotine enhances alcohol intake and dopaminergic responses through β2* and β4* nicotinic acetylcholine receptors

**DOI:** 10.1038/srep45116

**Published:** 2017-03-23

**Authors:** Stefania Tolu, Fabio Marti, Carole Morel, Carole Perrier, Nicolas Torquet, Stephanie Pons, Renaud de Beaurepaire, Philippe Faure

**Affiliations:** 1 Sorbonne Universités, UPMC Univ Paris 06, INSERM, CNRS, Neurosciences Paris Seine - Institut de Biologie Paris Seine (NPS - IBPS), 75005, Paris, France; 2 Groupe Hospitalier Paul Guiraud, BP 20065, F-94806, Villejuif, France; 3 Institut Pasteur, Unité Neurobiologie Intégrative des Systèmes Cholinergiques, Département de Neuroscience, F-75724, Paris, France; 4 CNRS, UMR 3571, F-75724, Paris, France

## Abstract

Alcohol and nicotine are the most widely co-abused drugs. Both modify the activity of dopaminergic (DA) neurons of the Ventral Tegmental Area (VTA) and lead to an increase in DA release in the Nucleus Accumbens, thereby affecting the reward system. Evidences support the hypothesis that distinct nicotinic acetylcholine receptors (nAChRs), the molecular target of acetylcholine (ACh) and exogenous nicotine, are also in addition implicated in the response to alcohol. The precise molecular and neuronal substrates of this interaction are however not well understood. Here we used *in vivo* electrophysiology in the VTA to characterise acute and chronic interactions between nicotine and alcohol. Simultaneous injections of the two drugs enhanced their responses on VTA DA neuron firing and chronic exposure to nicotine increased alcohol-induced DA responses and alcohol intake. Then, we assessed the role of β4 * nAChRs, but not β2 * nAChRs, in mediating acute responses to alcohol using nAChR subtypes knockout mice (β2−/− and β4−/− mice). Finally, we showed that nicotine-induced modifications of alcohol responses were absent in β2−/− and β4−/− mice, suggesting that nicotine triggers β2* and β4 * nAChR-dependent neuroadaptations that subsequently modify the responses to alcohol and thus indicating these receptors as key mediators in the complex interactions between these two drugs.

Alcohol and nicotine are the most commonly abused drugs in the world and their use triggers a broad range of serious negative health consequences with a high cost for the society^1^,^2^. Despite dissimilarities in their mechanisms of action and in their behavioural effects, alcohol and tobacco use commonly occur together. A very large majority (80–90%) of dependent drinkers smoke cigarettes^3^, and alcoholism has been estimated to be 10–14 times more common among smokers than non- smokers^4^.

Multiple factors may contribute to this high comorbidity, including environmental, psychosocial and genetic background^5^,^6^. Animal studies also point out the possibility of shared neurobiological mechanisms influencing the development of this co-addiction^7^. Indeed, among others, both alcohol and nicotine act on the mesocorticolimbic dopaminergic (DA) system. This system, originating in the Ventral Tegmental Area (VTA) of the midbrain and projecting to the Nucleus Accumbens (NAcc) and Prefrontal Cortex (PFC), is involved in reward, motivation, memory and cognition. Both systemic nicotine and alcohol increase synaptic DA release in the NAcc^8^, a key event that is considered to initiate reinforcement.

Increase in DA release is, for both drugs, mainly the consequence of an increase in VTA DA neuron firing rate and bursting activity^9^,^10^. However, if nicotine exerts its reinforcing effects by acting on nicotinic acetylcholine receptors (nAChRs), alcohol has been shown to act through a variety of neuronal receptors and ion channels, including different nAChR subtypes^11^. Several animal studies suggest a role for specific nAChRs in alcohol-elicited DA responses and alcohol reward^12^,^13^,^14^,^15^, thus making nAChRs a potential candidate for the molecular target of alcohol and nicotine interaction.

nAChRs are a family of pentameric ligand-gated ion channels made up of different α (α2-α10) and β (β2- β4) subunits which can assemble in multiple combinations^16^. In this paper, our interest focused on β2-containing (β2*) and β4 * nAChRs as they show specific DA phenotypes with regards to nicotine reinforcement. β2−/− mice lack the ability to self-administer nicotine and do not display nicotine-induced DA release in the NAcc^17^,^18^,^19^, while β4−/− mice have been recently implicated in the control of nicotine consumption^20^,^21^. The interest for β4*-nAChRs is also supported by human genetic studies assessing the implication of this receptor in the vulnerability to nicotine dependence and in the age of initiation for both tobacco and alcohol consumption^6^,^22^,^23^.

To investigate the multifaceted relationship between alcohol and nicotine, we used *in vivo* electrophysiology and an alcohol-drinking paradigm to analyse acute and chronic interactions between these two drugs. In parallel, we used transgenic mice to investigate the role of specific nAChRs in alcohol-induced responses and alcohol intake in nicotine-naïve and nicotine-exposed mice.

## Results

### Acute and chronic nicotine enhance responses to alcohol

**Figure 1:**
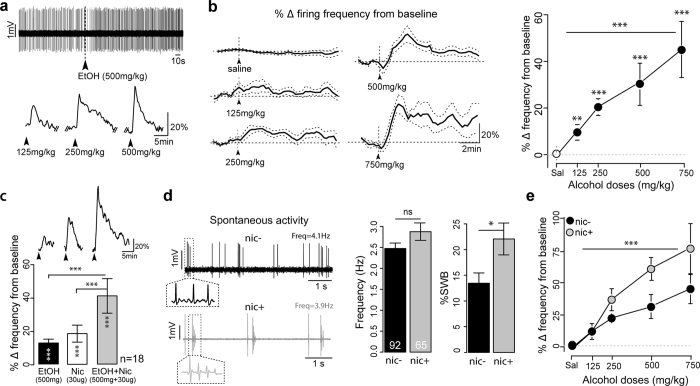
Acute and chronic nicotine enhance alcohol responses of VTA DA neurons. (**a**) (top) Typical electrophysiological recording showing the increase in firing rate of VTA DA cell induced by 500 mg/kg i.v. alcohol injection in WT mice. (bottom) Example responses of a VTA DA neuron to consecutive doses of alcohol. Arrowheads indicate the time of the injection. (**b**) (left) Mean ± SEM DA cell firing frequency modification after injection of saline and the indicated ethanol dose (saline, n = 28; 125 mg/kg, n = 13; 250 mg/kg, n = 14; 500 mg/kg, n = 14; 750 mg/kg, n = 7). Arrowheads indicate the time of the injection. (right) Dose-response curve of ethanol-elicited responses for the same groups of neurons. ***p < 0.001, **p < 0.01, Wilcoxon paired test from baseline. Horizontal lines indicate significant dose effect (one-way ANOVA). (**c**) (top) Example responses of a VTA DA neuron to consecutive injections of alcohol, nicotine and combined alcohol + nicotine. Arrowheads indicate the time of the injections. (bottom) Barplot of the maximum of firing frequency variation from baseline (mean ± SEM) after alcohol (black), nicotine (white), or combined alcohol + nicotine injections (gray). ***p < 0.001, Wilcoxon paired test from baseline is indicated within each vertical bar. Difference between groups is indicated above the horizontal lines. ***p < 0.001, Wilcoxon paired test between groups. (**d**) (left) Examples of electrophysiological recordings of the basal activity of a DA neuron of a nic− (black) and a nic+ (gray) mouse. Insets: Enlarged views of two or more action potentials. (right) Barplot of the mean frequency and %SWB for nic− (black, n = 92) and nic+ mice (gray, n = 65). *p < 0.05, Wilcoxon test. (**e**) Dose-response curve of ethanol-elicited DA cell responses for nic− (black) and nic+ (gray) mice. Mean ± SEM of variation from baseline in firing frequency. Horizontal lines indicate significant dose effect and vertical lines indicate treatment effect (***p < 0.001, *p < 0.5, two-way ANOVA. Nic−: saline: n = 36; 125 mg/kg: n = 19; 250 mg/kg: n = 22; 500 mg/kg: n = 17; 750 mg/kg: n = 9; nic+: saline: n = 19; 125 mg/kg: n = 3; 250 mg/kg: n = 9; 500 mg/kg: n = 9; 750 mg/kg: n = 12).

We first aimed to evaluate *in vivo* the effect of nicotine administration on the evoked response of VTA DA cells to i.v. injections of alcohol. Putative VTA DA neurons were identified according to their location and well-established electrophysiological and pharmacological criteria^9^,^17^ (see Methods). First, we characterized the evoked response of VTA DA cells to i.v. injections of alcohol. *In vivo*, alcohol injections resulted in a dose-dependent increase of the firing rate and of the bursting activity (%SWB, percentage of spike within a burst) of VTA DA neurons in C57BL/6 J mice (Fig. 1a,b and Fig. S1a) (∆Frequency: One-way ANOVA, F_(4, 72)_ = 10.9, p < 0.001; ∆%SWB: Kruskal-Wallis, X^2^ = 10.5, df = 4, p < 0.05). Similarly to what we observed with nicotine^24^, a fraction of VTA DA cells were inhibited by alcohol, but the current study focuses on cells that were excited by alcohol.

We then performed, on the same neurons, simultaneous injections of the two drugs and compared the responses with those obtained with a single injection of nicotine (30 μg/kg) or of alcohol (500 mg/kg) alone. Concomitant i.v. injections of nicotine and alcohol resulted in a change in the firing frequency of greater amplitude compared to the two drugs injected separately (Fig. 1c) (Paired Wilcoxon test: double vs single nicotine: V = 5, p < 0.001; double vs single alcohol: V = 13, p < 0.001). These data suggest that the two processes, i.e. alcohol- and nicotine-evoked responses, are not in competition and do not saturate the DA cell responses, at least at the tested doses. They are also in line with previous studies showing an additive and/or synergistic effects in the VTA to NAcc pathway^25^,^26^,^27^. Concurrent injections did not increase the %SWB further compared to single injections of each drug alone (Fig. S1b) (Paired Wilcoxon test: double vs single nicotine: V = 14, p = 1; double vs single alcohol: V = 18, p = 0.06). This could be explained by the fact that bursting modifications induced by alcohol are of smaller amplitudes compared to nicotine.

Given that tobacco addiction increases the incidence of alcohol abuse in humans, we then investigated the effect of chronic nicotine on alcohol-induced responses. Mice were exposed to chronic nicotine (10 mg/kg/d) using osmotic Alzet^®^ mini-pumps (MPs, see Materials and Methods) for 22–26 days. VTA DA cells spontaneous activity, alcohol-induced responses of DA neurons and alcohol intake were then estimated in the presence of chronic nicotine.

At a cellular level, chronic nicotine pre-exposure induced an increase in the spontaneous bursting activity (%SWB) of VTA DA neurons of mice (nic+) compared to control animals (nic−; a pooled group of naïve mice and mice with mini-pumps delivering saline, see SI and Fig. S2) (∆%SWB: W = 2650.5, p < 0.05; but no modification in the firing frequency (∆Frequency: t = −1.6, df = 113.9, p = 0.1) (Fig. 1d). It also increased the sensitivity of VTA DA neurons to acute injections of alcohol, as indicated by an upward shift of the dose-response curve for both the firing frequency and the %SWB (Fig. 1e and Fig. S1c) (∆Frequency: Two-way ANOVA: dose: F_(3, 92)_ = 10.0, p < 0.001; nicotine effect: F_(1, 92)_ = 8.2, p < 0.01; ∆%SWB: Kruskall-Wallis: nicotine effect: X^2^ = 6.7;df = 3, p < 0.01).

**Figure 2:**
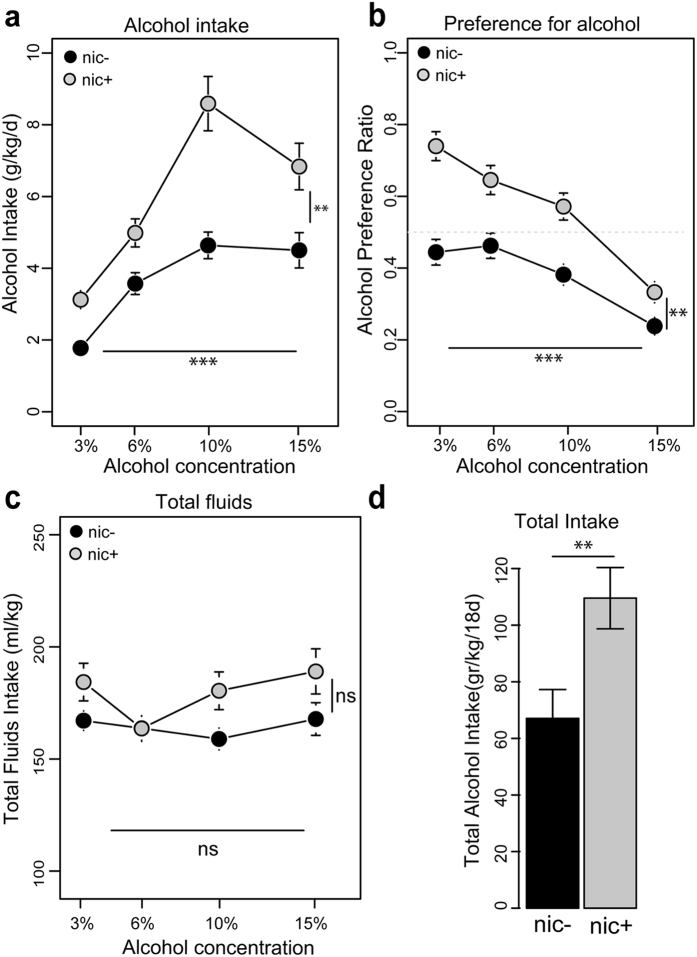
Chronic nicotine modifies alcohol intake. (**a**) Mean ± SEM of ethanol consumption (gr/kg) in nic− (black, n = 18) and nic+ (gray, n = 15) mice during the two bottle choice procedure. Horizontal lines indicate significant dose effect and vertical lines indicate treatment effect. ***p < 0.001, **p < 0.01, repeated measures two-way ANOVA. (**b**) Mean ± SEM of the preference ratio for alcohol over total fluid intake for the same groups of mice. ***p < 0.001, **p < 0.01, repeated measures two-way ANOVA; (**c**) Mean ± SEM of the total fluids intake for the same groups of mice. (**d**) Barplot of the total alcohol intake (within the 18 days procedure) for the same groups. **p < 0.01, Wilcoxon test.

At a behavioural level, this DA cell sensitization matched with an increase in alcohol consumption in a 24-hour voluntary alcohol-drinking paradigm (see Materials and Methods). In this paradigm, mice had to choose between water and increasing concentrations of alcohol during 18 consecutive days. Nic+ mice ingested higher quantities of alcohol compared to their control mice (nic−; Fig. 2a) (repeated measures ANOVA: dose: F_(3, 93)_ = 14.2, p < 0.001; nicotine effect: F_(1, 31)_ = 8.1; p < 0.01). Similarly, the preference ratio for ethanol was higher for nic+ than for nic− mice (Fig. 2b, dose: F_(3, 93)_ = 16.3, p < 0.001; nicotine effect: F_(1, 31)_ = 10.1, p < 0.01). Moreover, the total fluid intake (alcohol+water) was relatively constant across concentration (F_(3, 93)_ = 1.3; p = 0.3) and similar for the two groups (F_(1, 31)_ = 1.7; p = 0.2), thus suggesting that the increased consumption was not due to an increase in thirst, but rather to the increased rewarding properties of the drug (Fig. 2c). Finally, the total alcohol intake during the 18 day-procedure was greater for nic+ than nic− mice (Fig. 2d, Wilcoxon test: W = 59, p < 0.01). Such results highlighted an adaptation set up by nicotine pre-treatment and are consistent with previous behavioural studies that reported an increase in alcohol intake or preference after nicotine treatment^28^,^29^.

### Alcohol-induced firing frequency change is modified in β4−/−, but not in β2−/− mice

**Figure 3:**
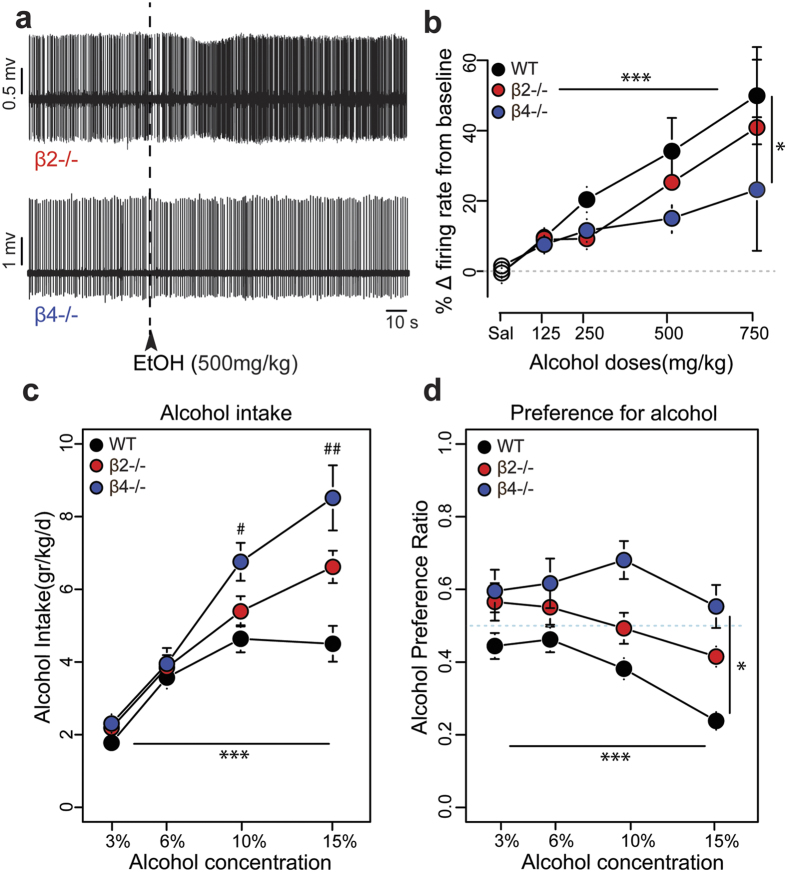
Alcohol-induced responses and alcohol reward are modified in β4−/−, but not in β2−/− mice. (**a**) Typical electrophysiological recordings showing the increase in firing rate of VTA DA cells induced by 500 mg/kg i.v. alcohol injection in β2−/− (top) and β4−/− mice (bottom). (**b**) Dose- response curves of ethanol-elicited DA cell responses for WT (black), β2−/− (red) and β4−/− mice (blue). Horizontal lines indicate significant dose effect and vertical lines indicate strain effect. ***p < 0.001, *p < 0.5, two-way ANOVA (see Results section for statistical details). WT: saline: n = 28; 125 mg/kg: n = 13; 250 mg/kg: n = 14; 500 mg/kg: n = 14; 750 mg/kg: n = 7; β2−/−: saline: n = 15; 125 mg/kg: n = 5; 250 mg/kg: n = 10; 500 mg/kg: n = 11; 750 mg/kg: n = 8; β4−/−: saline: n = 23; 125 mg/kg: n = 9; 250 mg/kg: n = 10; 500 mg/kg: n = 14; 750 mg/kg: n = 7. (**c**) Mean ± SEM of ethanol intake (gr/kg) in WT (black, n = 18), β2−/− (red, n = 9) and β4−/− mice (blue, n = 6) during the two bottle choice procedure. ***p < 0.001, repeated measures two-way ANOVA; ^##^p < 0.01, ^#^p < 0.05, multiple comparisons with Bonferroni correction. (**d**) Mean ± SEM of the preference ratio for alcohol over total fluid intake for the same groups of mice. ***p < 0.001; *p < 0.05, repeated measures two-way ANOVA (see Results section for statistical details).

The next step was to investigate the contribution of specific nAChRs in alcohol electrophysiological effects and alcohol intake. The spontaneous activity of DA neurons from β2−/− mice is characterized by a decrease in firing rate (t = 4.8, df = 169.3, p < 0.001) and in bursting activity (W = 2524, p < 0.001) compared to WT mice, whereas neurons from β4−/− mice display the same spontaneous activity (Frequency: t = −0.3, df = 166.2, p = 0.7; %SWB: W = 3567, p = 0.8) as WT mice (Fig. S3a,b and refs 9 and 21). Furthermore, VTA DA neurons from β2−/− mice did not respond to acute injections of nicotine (30 μg/kg) (W = 148, p = 0.9), whereas β4−/− mice still responded to the drug (W = 102, p < 0.001) (Fig. S3c and refs 9,17,19 and 21). In response to acute injections of alcohol (500 mg/kg), VTA DA neurons from both β2−/− and β4−/− mice displayed a dose-dependent increase of their firing rate (Fig. 3a). ANOVA analysis for the firing frequency modification including WT and both transgenic mice demonstrated a main effect of alcohol dose (Fig. 3b) (F_(3, 115)_ = 6.5; p < 0.001) and revealed a genotype effect (F_(2, 115)_ = 3.6, p < 0.05). Subsequent ANOVA between WT and β2−/− mice showed only a dose effect (F_(3, 75)_ = 5.1, p < 0.01), but no genotype (F_(1, 75)_ = 0.7, p = 0.2), nor dose-genotype effect (F_(3, 75)_ = 0.1, p = 0.9) was observed, thus indicating that DA neurons from β2−/− mice were not different from those of WT mice in their responses to alcohol. On the contrary, subsequent ANOVA between WT and β4−/− mice revealed a significant dose effect (F_(3, 81)_ = 4.8, p < 0.01) together with a genotype effect (F_(1, 81)_ = 4.8, p < 0.05), but no dose-genotype interaction (F_(3, 81)_ = 0.9, p = 0.4), showing that responses to alcohol were significantly reduced in β4−/− mice. Alcohol-induced %SWB variations in β2−/− and β4−/− mice were, contrarily to the variations in firing frequencies, not statistically different from those of WT mice (Fig. S1d) (Kruskal-Wallis: genotype effect: X^2^ = 1.6, df = 2, p = 0.4). This could again be explained by the small effect of alcohol on the bursting activity of DA neurons. Having characterized alcohol-elicited VTA DA responses, we tested both transgenic mice for alcohol consumption in comparison to WT mice (Fig. 3c,d). ANOVA between WT and both transgenic mice revealed a main effect of alcohol concentration (F_(3, 90)_ = 27.2; p < 0.001), no genotype effect (F_(2, 30)_ = 2.2; p = 0.13), but a dose-genotype interaction (F_(6, 90)_ = 2.1; p = 0.05). *Post hoc* analysis showed that β4−/− mice consumed significantly more alcohol than WT mice when highest alcohol concentrations were presented (10%: t = −3.3, df = 60.7, p < 0.05; 15%: t = −3.9, df = 47.7, p < 0.01), while β2−/− mice consumed similar amounts as WT mice (Fig. 3c). To further analyse this shift toward higher doses, we measured the intake modification switching from the dose of 10% to that of 15% in WT and mutant mice (Fig. S4a). We found that WT mice did not modify their intake when switching between these two doses (Wilcoxon paired test, 10% vs 15%: V = 2285, p = 0.3), thus reaching a *plateau* in their consumption. In contrast, β2−/− and β4−/− mice kept increasing their consumption (Wilcoxon paired test, 10% vs 15%: β2−/−: V = 340.5, p < 0.05; β4−/−: V = 112, p < 0.05). The comparison of the intake modifications between the three groups revealed a statistical difference between WT and β4−/− mice (Wilcoxon: W = 921, p < 0.05) but only a tendency between WT and β2−/− mice (Wilcoxon: W = 1558, p = 0.07), thus confirming a role of β4nAChRs in mediating the acute effects of alcohol. Moreover, the preference ratio for alcohol was also modified in transgenic mice (Fig. 3d). ANOVA comparing the three genotypes showed a main significative dose effect (F_(3, 90)_ = 8.2, p < 0.001), a significative genotype effect (F_(2, 30)_ = 2.6, p < 0.05) but no dose-genotype interaction (F_(6, 90)_ = 0.8, p = 0.5). Subsequent ANOVA between WT and β2−/− mice revealed only a significative dose effect (F_(3, 75)_ = 8.1, p < 0.001), but no genotype effect (F_(1, 25)_ = 1.7, p = 0.1) nor dose-genotype interaction (F_(3, 75)_ = 0.3, p = 0.09). ANOVA between WT and β4−/− mice revealed a significative dose effect (F_(3, 66)_ = 7.4, p < 0.001), a significative genotype effect (F_(1, 22)_ = 7.4, p < 0.05), and no dose-genotype interaction (F_(3, 66)_ = 1.5, p = 0.2).

Thus, these results demonstrated that, compared to WT mice, β4−/−, but not β2−/− mice displayed both a modified VTA DA cells evoked response to alcohol and a modified alcohol drinking profile.

### Chronic nicotine has no effect on alcohol-elicited responses and alcohol intake in β2−/− and β4−/− mice

We then addressed the question of the impact of chronic nicotine exposure on the alcohol response in these mutant mice. Indeed, chronic exposure to nicotine induces a series of adaptations that particularly implicate heteromeric nAChRs^30^. We thus asked whether the enhanced responses to alcohol induced by chronic nicotine exposure might rely on modifications of the expression or function of nAChRs in the DA system and thus analysed alcohol-elicited responses in β2−/− and β4−/− mice after chronic exposure to nicotine (β2nic+ and β4nic +mice).

**Figure 4:**
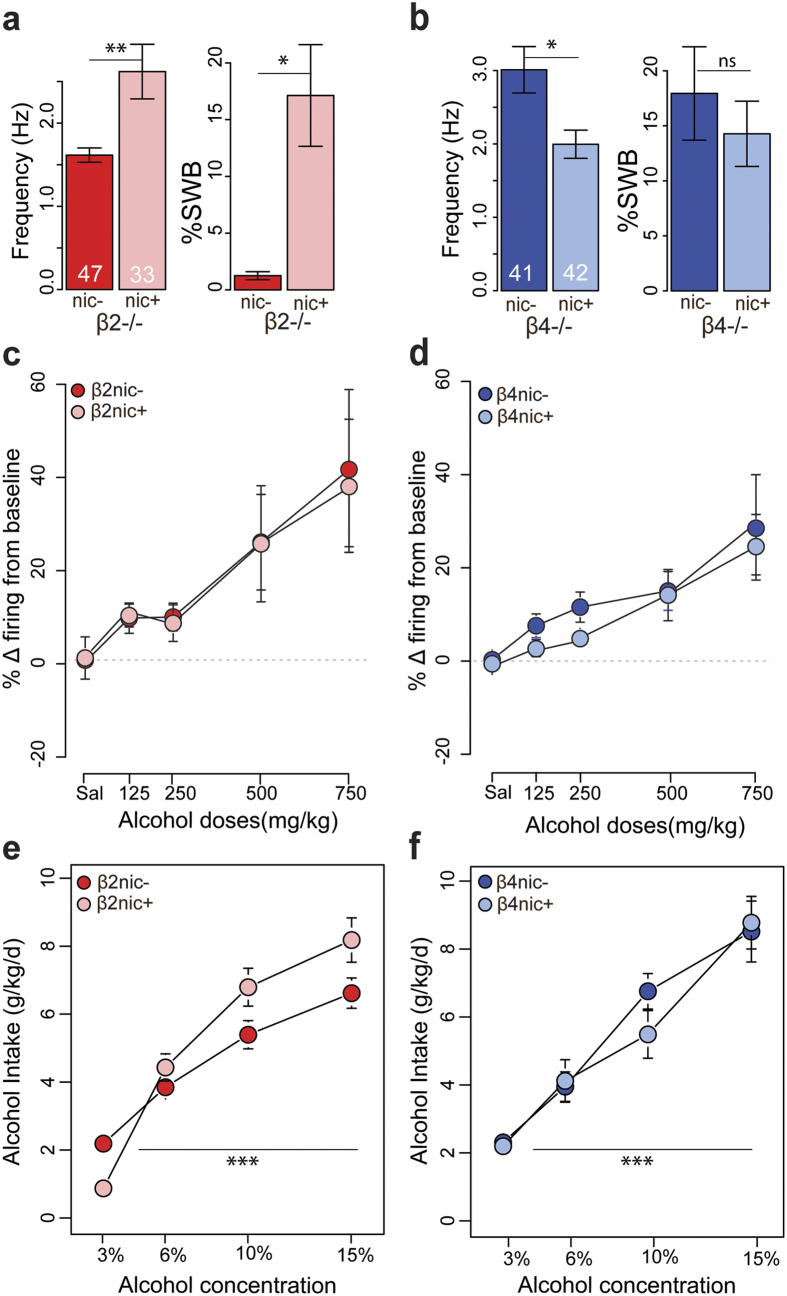
Alcohol-elicited responses and alcohol intake of β2−/− and β4−/− mice are not modified by chronic nicotine. (**a**) Barplot of the mean frequency and %SWB for β2nic− (red, n = 47) and β2nic+ mice (pink, n = 33). **p < 0.01, t-test; *p < 0.05, Wilcoxon test. (**b**) Barplot of the mean frequency and % SWB for β4nic− (blue, n = 41) and β4nic+ mice (light blue, n = 42). *p < 0.05, t-test. (**c**) Dose- response curves of ethanol-elicited DA cell responses for β2nic− (red) and β2nic+ (pink). β2nic−: saline: n = 15; 125 mg/kg: n = 5; 250 mg/kg: n = 10; 500 mg/kg: n = 11; 750 mg/kg: n = 8; β2nic+: saline: n = 12; 125 mg/kg: n = 6; 250 mg/kg: n = 7; 500 mg/kg: n = 10; 750 mg/kg: n = 5. (**d**) Dose- response curves of ethanol-elicited DA cell responses for β4nic− (blue) and β4nic+ (light blue). β4nic−: saline: n = 23; 125 mg/kg: n = 9; 250 mg/kg: n = 10; 500 mg/kg: n = 14; 750 mg/kg: n = 7; β4nic+: saline: n = 15; 125 mg/kg: n = 7; 250 mg/kg: n = 10; 500 mg/kg: n = 8; 750 mg/kg: n = 6. (**e**) Mean ± SEM of ethanol consumption (gr/kg) in β2nic− (red, n = 9) and β2nic+ (pink, n = 8) mice during the two bottle choice procedure. (**f**) Mean ± SEM of ethanol consumption (gr/kg) in β4nic− (blue, n = 6) and β4nic+ (light blue, n = 6) mice during the two bottle choice procedure.

Surprisingly, in basal conditions, we found that, compared to their respective nic− controls, β2nic+ mice displayed an increased basal firing rate and bursting activity (∆Frequency: t = −2.9, df = 36.4, p < 0.01; ∆%SWB: W = 558.5, p < 0.05) (Fig. 4a), whereas β4nic+ mice showed a decreased firing rate and an unchanged bursting activity (∆Frequency: t = 2.7, df = 66.2, p < 0.01; ∆%SWB: W = 862, p = 0.9) (Fig. 4b). However, when we compared the responses evoked by alcohol in nic− and in nic+ mice of the two transgenic mouse lines, we observed no modification neither in the variation of the firing frequency (Fig. 4c,d), nor in the variation of %SWB (Fig. S1e,f). VTA DA neurons of β2nic+ and β4nic +mice (unlike those of WTnic+ mice) showed responses to alcohol of the same amplitude as β2nic− and β4nic− mice, respectively (∆Frequency: ANOVA: β2nic− vs β2nic+: dose effect: F_(4, 78)_ = 4.9, p < 0.01; nicotine effect: F_(1, 78)_ = 0.5, p = 0.5; dose × nicotine effect: F_(4, 78)_ = 0.5, p = 0.7; β4nic− vs β4nic+: dose effect: F_(4, 99)_ = 8.0, p < 0.001; nicotine effect: F_(1, 99)_ = 2.6; p = 0.1; dose × nicotine effect: F_(4, 99)_ = 0.2, p = 0.9; ∆%SWB: Kruskal-Wallis: β2nic− vs β2nic+, nicotine effect: X^2^ = 0.01, df = 1, p = 0.9; β4nic− vs β4nic+, nicotine effect: X^2^ = 0.7, df = 1, p = 0.4). Similarly, β2nic and β4nic mice failed to increase alcohol consumption, showing the same intake profiles as their nic− controls (Fig. 4e,f) (β2nic− vs β2nic+: dose effect: F_(3, 45)_ = 33.9, p < 0.001; nicotine effect: F_(1, 15)_ = 0.6, p = 0.4; dose × nicotine effect: F_(3, 45)_ = 2.3; p = 0. 1; β4nic− vs β4nic+: dose effect: F_(3, 30)_ = 25; p < 0.001; nicotine effect: F_(1, 10)_ = 0.1; p = 0.7; dose × nicotine effect: F_(3, 30)_ = 0.5; p = 0.7). Interestingly, β2nic+ and β4nic+ kept showing the same increase in alcohol intake when switching from the dose of 10% to that of 15%, contrary to WT nic+ mice, for which the amount of alcohol consumed at the dose of 15% was not statistically different from the intake at the dose of 10% (Fig. S4b) (Wilcoxon paired test, 10% vs 15%: WT nic+: V = 1647, p = 0.2; β2nic+: V = 203, p < 0.01; β4nic+: V = 87, p < 0.01). However, it is important to note that these effects are not dependent on nicotine treatment, since they were already expressed in basal conditions, thus in absence of nicotine.

To summarize, in these mutant mice, despite an effect on the spontaneous VTA DA cells activity, chronic nicotine exposure does not increase VTA DA cells response nor alcohol intake. These results thus suggest that the lack of enhanced responses to alcohol or alcohol intake resulted from the absence of neuroadaptations at the level of β2 and/or β4nAChRs expression and/or function that occurred during nicotine exposure in WT mice.

## Discussion

The nicotine-alcohol interactions underlying the high incidence of co-addiction are a very complex phenomenon for which several mechanisms have been proposed. We focused our attention on the mesolimbic DA system that is a potential substrate for mechanistic interaction between these two drugs^7^,^8^,^31^. We first studied the electrophysiological responses of VTA DA neurons to different doses of alcohol in order to establish the dose response curve in C57BL/6J WT mice. To evaluate the short-term interactions between alcohol and nicotine we performed concurrent injections of both drugs and compared them to the responses of each drug alone. A simultaneous injection of nicotine and alcohol led to a summation of the individual effects. Our data are in line with previous *in vitro* and *in vivo* studies showing additive and/or synergistic effects in the VTA to NAcc pathway^25^,^26^,^27^,^32^,^33^ and further supports the hypothesis that combined effects of nicotine and alcohol on the DA system may contribute to the high incidence of co-abuse. Moreover, this cumulative effect indicates that, at least at the tested doses, the effect of one does not occlude the other and, more importantly, that the two drugs may cooperate to enhance DA transmission increasing the sensation of pleasure and reward.

One of the possible factors contributing to the development of drug co-abuse is the cross-sensitization, in which the chronic use of one drug induces sensitization to the other. To address this question, we investigated the effects of chronic nicotine pre-exposure on the evoked responses of DA cells to alcohol and on alcohol intake. To achieve a constant level of nicotine, we chose a continuous mode of administration that avoids the need of repetitive injections. Continuous infusion of nicotine has been previously shown to induce behavioural and molecular adaptations, including nAChRs upregulation^34^,^35^,^36^,^37^,^38^, which is considered an important feature of nicotine dependence^39^. Our results showed that chronic nicotine increases the basal bursting activity of DA neurons in WT mice. Previous studies investigating the effects of a passive chronic infusion of nicotine in rodents reported divergent results, showing either no effect of nicotine treatment on the bursting activity or a decrease in the firing rate of DA cells^36^,^40^,^41^,^42^. Dissimilar data can be explicated by diversities in nicotine regimen. For example, Besson *et al*.^36^ and Tan *et al*.^40^ used a different nicotine dose or treatment duration, whereas in Caillé *et al*.^41^ and in Grieder *et al*.^42^, MPs were removed 16 or 24 hours before the electrophysiological recordings, thus at the time when animals were already experiencing motivational withdrawal from nicotine. In the same paper, Caillé *et al*. also reported that voluntary nicotine self-administration, but not passive exposure, induced an increase in both the firing rate and bursting activity of VTA DA cells. These adaptations are in part mediated by the BNST (Bed Nucleus Stria Terminalis) glutamatergic drive onto the VTA, which has been suggested to be implicated in learning processes and memory. Nevertheless, the potentiation of glutamatergic signalling on VTA DA cells is not the sole mechanism accounting for bursting activity of DA neurons. We have shown in a previous paper^17^ that the cholinergic modulation (through β2nAChRs) of GABA neurons is necessary for the bursting activity of DA neurons. In addition, it has been reported, that passive nicotine infusion through MPs induce an upregulation of nAChRs, in particularly α4β2nAChRs expressed on GABA neurons^37^. In this context, we could speculate that, in mice and at this dose, passive nicotine induces nAChR upregulation, sufficiently to enhance the bursting activity of DA neurons, while the association between active responding and reward delivery is necessary to strengthen glutamatergic inputs from the BNST on DA cells. In addition to DA cell spontaneous firing, chronic nicotine also potentiated VTA DA cell responses to alcohol in WT animals. Such results highlight a neural adaptation, set up by nicotine pre-treatment, which affect alcohol responses and are in line with microdialysis studies showing an increase in alcohol-evoked DA release in the NAcc after repeated nicotine exposure^43^,^44^. More interestingly, the sensitization of VTA DA neurons concurred with a sensitization of the animal to the rewarding properties of ethanol, demonstrated by an increased intake. Our results are thus in accordance with previous behavioural studies using different self-administration paradigms which report an increase in alcohol intake or preference after nicotine treatment^28^,^29^,^45^.

Given the increasing evidences suggesting nAChRs as a common molecular substrate for alcohol and nicotine interaction^12^,^13^,^14^,^15^,^25^,^26^, we tested β2−/− and β4−/− mice for alcohol responses in order to assess their involvement in alcohol action on the reward system. We found that β2*nAChRs are not required for the acute effects of alcohol, given that VTA DA cells of β2−/− mice showed comparable responses to those of WT mice, and that β2−/− mice ingested unchanged amounts of alcohol. These findings are in agreement with the alcohol-drinking phenotype of β2−/− mice^46^ and confirm studies showing that selective blockade of β2*nAChR affects neither alcohol consumption^12^,^15^ nor alcohol-elicited DA release in the NAcc^47^,^48^. Interestingly, our data unveil a key role for β4*nAChRs in mediating alcohol responses and in modulating its reinforcing properties, thereby defining the sensitivity of the reward system to alcohol. This result contrasts with a recent study showing that β4−/− mice consumed similar amount of alcohol as WT mice^49^. But this study not only differed for the concentrations of alcohol presented, but it used a completely different paradigm for alcohol access, the drinking-in-the-dark (DID) procedure, which is a model of binge drinking leading to high alcohol consumptions^50^. The inverted relationship between VTA DA system sensitivity and drug self-administration that we observed in β4−/− mice has been already described for α5−/− mice for nicotine^51^, where the decreased sensitivity of DA cells to nicotine was paralleled by a consumption shift to high doses. Deletion of β4*nAChRs, which was shown to result in an increased sensitivity for nicotine^21^, results here in an opposite, decreased sensitivity for alcohol. β4*nAChR subtype thus seems to be implicated, in both alcohol and nicotine responses, leading to a modification of the drug consumption. The downward shift of the dose-response curve of DA neurons found in β4−/− could reflect i) a decreased sensitivity to the reinforcing properties of alcohol so that higher doses are needed to experience the pleasurable effect of alcohol or ii) a decreased sensitivity to the aversive effects. In this latter hypothesis, β4nAChRs could play a regulatory role acting as a “brake” in mediating the negative effects of alcohol, so that mice lacking β4nAChRs may experience fewer signs of aversive effects, which may facilitate alcohol intake at high doses.

The neuronal mechanisms for alcohol and nicotine interaction are not fully understood, yet we clearly show here that they involve the DA system and β2 and β4*nAChRs. Indeed both drugs induce similar responses on DA neurons and their effects are amplified when injected together. Our work also reveal β4*nAChRs as possible actors implicated in the mediation of the acute effects of alcohol in the VTA, since the lack of this subunit modifies alcohol-evoked responses. Furthermore, it is well known that chronic nicotine exposure triggers a series of changes in nAChRs (distribution, stoichiometry or conformational state) but also various forms of synaptic plasticity, that outlast the presence of the drug and lead to the remodelling of neuronal circuits^52^. Among others, these adaptations underpin (i) modifications of VTA DA cells spontaneous activity and (ii) sensitization in VTA DA neuron responses to alcohol and in alcohol drinking behaviours. Our results suggest that nAChRs contributed to these two phenomena and that they seem to be independent. Indeed, despite chronic nicotine failed to induce any sensitization in DA responses to alcohol and in alcohol drinking behaviours in both β2−/− and β4−/− mice, it impacted VTA DA cells spontaneous activity. Yet, the level and the role of nicotine-induced neuroadaptations need further investigation, notably to elucidate whether cross-sensitization is due to molecular changes on nAChRs or rather to synaptic plasticity and to the consecutive reorganisation of DA circuits. Understanding this latter point will be of utmost importance to identify specific molecular targets for the development of more effective pharmacological treatments against alcohol and tobacco addiction.

## Materials and Methods

### Animals

Adult (aged 8–16 weeks) male C57BL/6J wild-type (WT), β2−/−18 and β4−/− mice^53^ were used in this study. Both constitutive KO mice were backcrossed onto C57BL/6J background for at least 20 generation and bred in the same life conditions at Charles River (L’Arbresle, France). Experiments were performed after at least one week of habituation in our animal facility. Animal care and experiments were conducted in accordance with European Ethical Committee guidelines and approved by the Charles Darwin Animal Experimentation Ethical Committee.

### *In vivo* electrophysiology

Single unit extracellular recordings were performed in anesthetized WT, β2−/− and β4−/− mice as detailed in the Supplementary Information. Briefly, glass electrodes containing 1.5% neurobiotin in 0.5% sodium acetate were lowered in the VTA according to stereotaxic coordinates derived from mouse brain atlas, and corrected empirically (antero-posterior: −3 to −4 mm; medio-lateral: 0.3 to 0.7 mm; dorso-ventral: −4 to −4, 8 mm from bregma). Electrical signals were amplified by a high-impedance amplifier (Axon Instruments) and monitored audibly through an audio monitor (A.M. Systems Inc.). The signal was digitized, sampled at 25 kHz and recorded on a computer using Spike2 software (Cambridge Electronic Design) for later analysis. To distinguish DA from non-DA neurons the following parameters were used: 1) regular firing rate; 2) firing frequency between 1 and 10 Hz; 3) action potential duration between the beginning and the negative trough superior to 1.1 ms. Intravenous injections of nicotine (30 μg/kg) and alcohol (125 mg/kg, 250 mg/kg, 500 mg/kg and 750 mg/kg) in the saphenous vein were performed in a final volume ranging from 20 to 120 μl, as function of the administered dose (See SI for drugs specifications). Nicotine dose was chosen according to previous studies showing that nicotine can be intravenously self-administered at this dose in mice and on the base of our previous works^17^,^19^,^24^,^54^. The range of alcohol doses injected were chosen on the base of previous *in vivo* electrophysiological studies in rats^10^. When possible, neurons were electroporated to allow neurobiotin internalization and labelling for neuron identification. D2 receptors pharmacology was performed on the last neuron of the experimental day (see SI and Fig. S5a,b).

### Two bottle choice procedure

24 hours voluntary drinking behaviour was carried out as specified in SI. Briefly, mice were offered water versus increasing concentrations of ethanol (3, 6, 10 and 15% (v/v)) within 18 days.

### Osmotic mini-pumps

Surgical implantation of mini-pumps containing nicotine (10 mg/kg/d) or saline solution (0.9% NaCl) is described in SI. This dose was chosen on the base of previous works showing that in mice such dose correspond to nicotine plasma levels comparable to those sampled in smokers, generally ranged from 10 to 50 ng/ml^35^,^55^.

### Statistical analysis

The analyses were led using the R software (http://www.r-project.org). Firing frequency was quantified over 60 s periods, with a 45 s overlapping period. Percentage of spikes within bursts (%SWB) corresponds to the percentage of spikes discharged within bursts in a given time interval. A two-sample t-test was used to compare mean firing rate in two populations while a non- parametric Wilcoxon test was used for %SWB. Shapiro tests were used to test the normality of the data. Firing frequency response was quantified as a percentage of variation from baseline on a 3- min period before and after injection and means were calculated within each dose and each group. One-way or two-way ANOVAs were used to analyse dose-response curves and differences between groups. %SWB variation was calculated as a percentage of variation from baseline on a 3- min period before and after injection. A Kruskal-Wallis test was used to analyse dose-response curves and differences between groups. For alcohol intake and alcohol preference two-way ANOVA with repeated measures were used, followed by Bonferroni tests for *post hoc* analysis, when applicable.

## Additional Information

**How to cite this article:** Tolu, S. *et al*. Nicotine enhances alcohol intake and dopaminergic responses through β2* and β4* nicotinic acetylcholine receptors. *Sci. Rep.* **7**, 45116; doi: 10.1038/srep45116 (2017).

**Publisher's note:** Springer Nature remains neutral with regard to jurisdictional claims in published maps and institutional affiliations.

## Supplementary Material

Supplementary Information
