# Supplementary material for: Nicotine enhances alcohol intake and dopaminergic responses through β2* and β4* nicotinic acetylcholine receptors
Source: Sci Rep. 2017 Mar 23;7:45116. doi: 10.1038/srep45116 (PMC5362818; doi:10.1038/srep45116)
Supplement: Supplementary Information [file srep45116-s1.pdf]

Supplementary Information

Nicotine enhances alcohol intake and dopaminergic responses through β2 and β4nAChRs.

Stefania Tolu (PhD) [1], Fabio Marti (PhD) [1], Carole Morel (PhD) [1,5], Carole Perrier (MSc) [2], Nicolas Torquet (MSc) [1], Stephanie Pons (MSc) [3,4], Renaud de Beaurepaire (PhD, MD) [2] and Philippe Faure (PhD) [1*]

[1] Sorbonne Universités, UPMC Univ Paris 06, INSERM, CNRS, Neurosciences Paris Seine - Institut de Biologie Paris Seine (NPS - IBPS), 75005 Paris, France

[2] Groupe Hospitalier Paul Guiraud, BP 20065, F-94806 Villejuif, France

[3] Institut Pasteur, Unité Neurobiologie Intégrative des Systèmes Cholinergiques, Département de Neuroscience, F-75724 Paris, France

[4] CNRS UMR 3571, F-75724 Paris, France

[5] Present address: Department of Pharmacology and Systems Therapeutics, Icahn School of Medecine at Mount Sinai, New York, NY, USA

*Correspondence:

Dr. Philippe Faure
Neuroscience Paris Seine NPS,
CNRS UMR8246, INSERM U1130
Université Pierre et Marie Curie UM 119,
Equipe NeuroPhysiologie et Comportement, Bat B, 5° étage,
Université Pierre et Marie Curie, Boite 16, 9 Quai St Bernard 75005 Paris, France
Tel: 00 33 1 44 27 39 40
Email: phfaure@gmail.com

Supplementary Information: Methods

Drugs

For electrophysiological recordings, alcohol was prepared as a 20% (v/v) solution in a sterile 0.9% NaCl solution from Ethanol 96% (EMPROVE® exp Ph Eur, BP, Merck Millipore) and injected in a volume proportional to the dose (20 to 120µl). Injected doses were: 125mg/kg, 250mg/kg, 500mg/kg and 750mg/kg. Nicotine hydrogen tartrate salt (Sigma-Aldrich) was freshly dissolved in sterile 0.9% NaCl solution and pH adjusted to 7.4 with NaOH and injected at the dose of 30 µg/kg (free base). Before drugs administration, control saline injections were performed in different final volumes (10, 40 and 120 µl). As no statistical difference was found between saline injections at different volumes solution (t.test: 10 vs 40: t=-0.2, df=22.5, p=0.8; 10 vs 120: t=0.03, df=13.8, p=0.9; 40 vs 120: t=0.2, df=13.5, p=0.8) (example for WT mice is shown in Fig. S5c), data were pooled and used as unique control injection. For pharmacological neuron identification, quinpirole hydrochloride and eticlopride hydrochloride (Tocris Bioscience), respectively the agonist and antagonist of dopaminergic D2 receptors, were dissolved in sterile 0.9% NaCl and intravenously injected at the dose of 1mg/kg in a final volume of 10µl.

In osmotic mini-pumps, nicotine was infused at the dose of 10mg/kg/d (free base). For intake experiment, alcohol-drinking solutions were presented as 3, 6, 10 and 15% (v/v) solutions in mineral water.

***In vivo* electrophysiology**

8-16 weeks old male (25 to 30 g) C57Bl/6J wild-type (WT), β2-/- and β4-/- mice were deeply anaesthetized with chloral hydrate (8%), 400 mg/kg i.p., supplemented as required to maintain optimal anesthesia throughout the experiment. The scalp was opened and a whole was drilled in the skull above the location of the VTA. The saphenous vein was catheterized for

intravenous administration of drugs. Extracellular recording electrodes were constructed from 1.5 mm O.D. / 1.17 mm I.D. borosilicate glass tubing (Harvard Apparatus) using a vertical electrode puller (Narishige). Under microscopic control, the tip was broken to obtain a diameter of approximately 1 μm. The electrodes were filled with a 0.5% NaCl solution containing 1.5% of neurobiotin tracer (AbCys) yielding impedances of 6-9 MΩ. Electrical signals were amplified by a high-impedance amplifier (Axon Instruments) and monitored audibly through an audio monitor (A.M. Systems Inc.). The signal was digitized, sampled at 25 kHz and recorded on a computer using Spike2 software (Cambridge Electronic Design) for later analysis. The electrophysiological activity was sampled in the central region of the VTA (coordinates: between 3.1 to 4 mm posterior to Bregma, 0.3 to 0.7 mm lateral to midline, and 4 to 4.8 mm below brain surface). Individual electrode tracks were separated from one another by at least 0.1 mm in the horizontal plane. Spontaneously active DAergic neurons were identified on the basis of previously established electrophysiological criteria (see main text, methods section). After a baseline recording of at least 5 minutes, a saline solution (0.9% sodium chloride) was injected into the saphenous vein, and after another 5 minutes, injection of alcohol and/or nicotine hydrogen tartrate were administered *via* the same route. Successive injections were performed after the neuron returned to its baseline, or when the firing activity returned stable for at least 3 minutes. For combined alcohol + nicotine injections, the two saphenous veins were catheterized and the two drugs concomitantly injected in the two veins. D2 receptors pharmacological identification was performed on the last neuron of the day (Supplementary Fig. S5b). Quinpirole hydrochloride in saline solution (1mg/kg) was injected intravenously, followed 5 minutes later by eticlopride hydrochloride (1mg/kg). Once D2-R pharmacology was applied, no further neurons were recorded and the animal was discarded.

Immunocytochemical identification of recorded neurons

When possible, neurons were electroporated and neurobiotin was expulsed from the electrode using positive current pulses as already described (Eddine *et al*, 2015). The mouse was then killed and the brain post-fixed in 4% paraformaldehyde. 60µm slices were cut on a vibratome. Fluorescence immunohistochemistry was performed as follows: free-floating VTA brain sections were incubated 1 hour at 4°C in a blocking solution of PBS containing 3% BSA (Bovine serum albumine) and 0.2% Triton X-100 and then overnight at 4°C in PBS containing primary antibodies at appropriate dilution, 1,5% BSA and 0.2% Triton X-100. The next day sections were rinsed with PBS and then incubated 3 hours at room temperature with secondary antibodies in a solution of 1,5% BSA and 0.2% Triton X-100 in PBS. After three rinses in PBS, slices were wet-mounted using Prolong Gold Antifade Reagent (Invitrogen).

To stain DAergic neurons and fibers, a mouse anti-Tyrosine Hydroxylase (aTH, Sigma) was used at 1:200 dilution. All secondary antibodies were used at 1:200 dilution: CY3-coniugated anti-mouse IgG and AMCA-coniugated anti-mouse IgG (Jackson Immunoresearch). An example of immunostained neurons is presented in Supplementary Fig. S5a.

Two bottle choice procedure

Mice were housed individually in standard cages equipped with two bottles, containing either alcohol or mineral water, to which they had continuous free access. Each bottle was attached to a precision weighing sensor mounted to the cage lid and interfaced to a computer that automatically measured the amount of liquid consumed over time (TSE systems, Germany). Quantity of alcohol or water ingested was recorded every minute. Food (standard pellets for rodents) was available *ad libitum.* Mice were subjected to a 4-days habituation period in which only water was presented in the two bottles. From the 5th day, mice were exposed to progressively increasing concentrations of ethanol (within 18 days) under the free-choice

procedure adapted from Kelaï *et al.* (2008). Mice were offered 3% ethanol (v/v) versus water for 4 days, then 6% ethanol for the next 4 days, then 10% ethanol for the next 5 days and finally animals had access to 15% ethanol for the last 5 days. Mice were weighed every 4 days and bottles positions changed every two or three days to control for position preference.

Surgical implantation of mini-pumps

Mice were slightly anesthetized with a ketamine (1.5%) and xylazine (0.05%) combination in PBS. Alzet® osmotic mini-pumps (mod. 2004; release rate: 0,25µL/h; duration 28±2 days) were implanted subcutaneously (s.c.) between the two scapulae, parallel to the spine. They delivered nicotine (10mg/kg/d) or vehicle (saline solution). Electrophysiological experiments were carried out between the 22nd and the 26th day after the implantation, thus before the end of the delivery duration to avoid the appearance of withdrawal or abstinence.

For the two bottle choice experiment under chronic nicotine, mice were implanted with MPs and housed in standard home cages. The 26th day of delivery MPs were removed and replaced with new MPs containing the same dose of nicotine (or saline) and animals were housed individually for the drinking experiment.

Control (nic-) group definition

For electrophysiological studies, the nic- DAergic neurons are a pool of cells from nicotine-naïve mice and mice implanted with MPs delivering saline solution, which showed similar spontaneous firing patterns and responses to alcohol (Supplementary Fig. S2). In WT mice, spontaneous activity: naïve WT mice (n=76, firing frequency: 2.51± 0.16 Hz and %SWB: 13.80 ± 2.15% (mean ±S.E.M.)) vs WTmpSal mice (n=27, firing frequency: 2.35± 0.19 Hz and %SWB: 12.43 ± 2.60% (mean ±S.E.M.)) p=0.53 (Welch Two Sample t-test) and p=0.68 (Wilcoxon rank sum test) for firing frequency and %SWB, respectively (Fig S2a). For ethanol

responses: ΔFrequency: Two-way ANOVA: dose: $F_{(3,\ 59)}=4.5$, $p<0.01$; group effect: $F_{(1,\ 59)}=0.01$, $p=0.9$; interaction dose x group : $F_{(3,\ 59)}=0.4$, $p=0.7$ (Fig S2b, c).

For the two-bottle choice paradigm, the nic- (Fig. 2a, b, c d), β2nic- and β4nic- (Fig. 4e, f) groups correspond to respectively WT, β2-/- and β4-/- mice receiving saline solution through mini-pumps.

REFERENCES

1. Eddine R *et al*. A concurrent excitation and inhibition of dopaminergic subpopulations in response to nicotine. *Scientific Reports,* ***5:*** 8184 (2015).

2. Kelaï S *et al*. Chronic voluntary ethanol intake hypersensitizes 5-HT(1A) autoreceptors in C57BL/6J mice. *Journal of Neurochemistry,* ***107:*** 1660–1670 (2008).

Supplementary Figure1

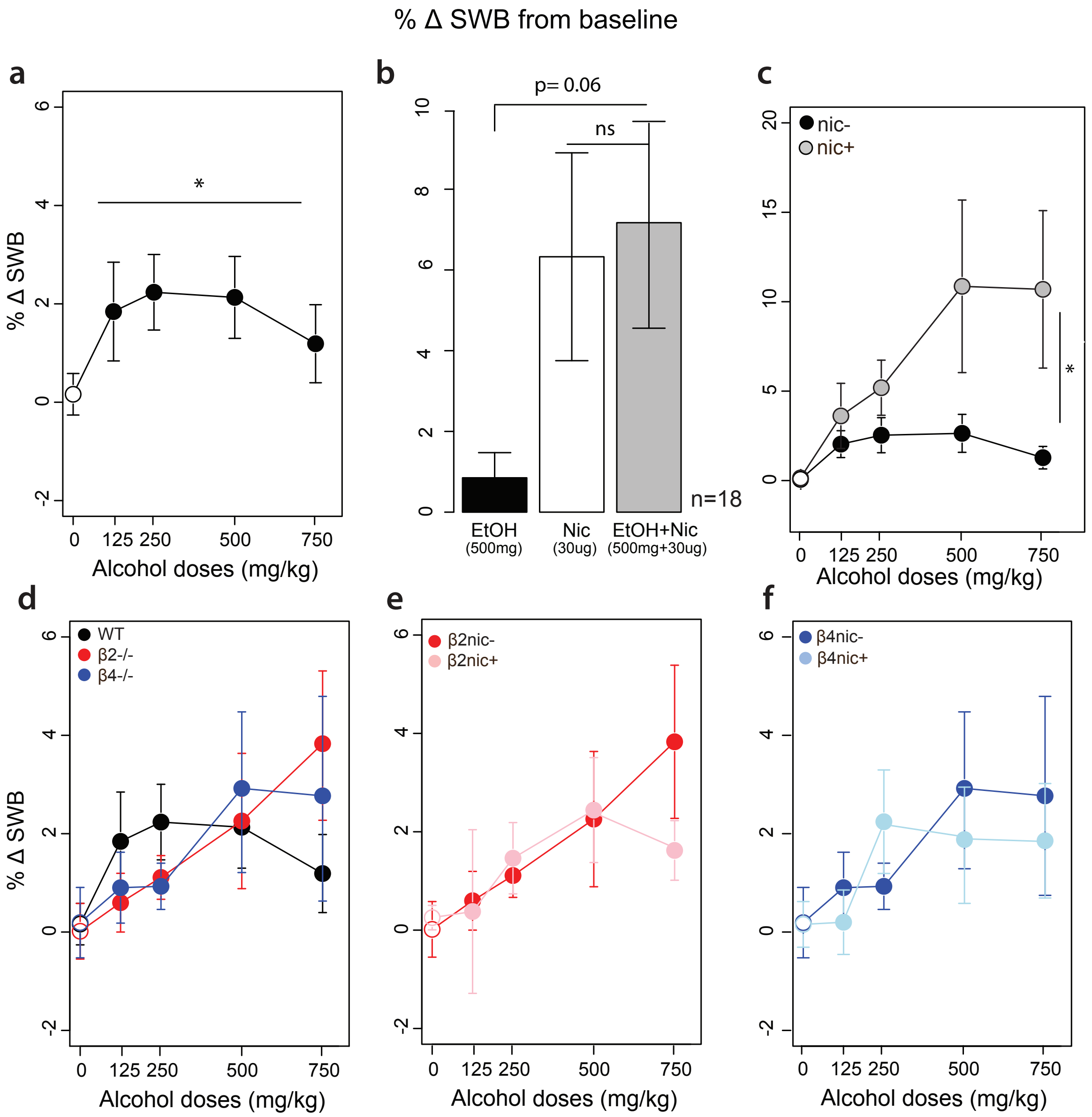

Supplementary Figure S1: Analyses of % SWB variation. (a) Mean ± SEM of variation from baseline in %SWB induced by alcohol injections (125, 250, 500 and 750 mg/kg) in WT mice. Horizontal line indicates significant dose effect (ANOVA, *$p<0.05$). (b) Mean ± SEM of variation from baseline in %SWB after an injection of alcohol (black), nicotine (white) or both drugs together (gray). (c) Mean ± SEM of variation from baseline in %SWB induced by alcohol injections in nic- (black) and nic+ (gray) WT mice. Vertical line indicates significant group effect (ANOVA, *$p<0.05$). (d) Mean ± SEM of variation from baseline in %SWB induced by alcohol injections in WT (black), β2-/- (red) and β4-/- (blue) mice. (e) Mean ± SEM of variation from baseline in %SWB induced by alcohol injections in β2nic- (red) and β2nic+ (pink) mice. (f) Mean ± SEM of variation from baseline in %SWB induced by alcohol injections in β4nic- (blue) and β4nic+ (lightblue).

Supplementary Figure 2

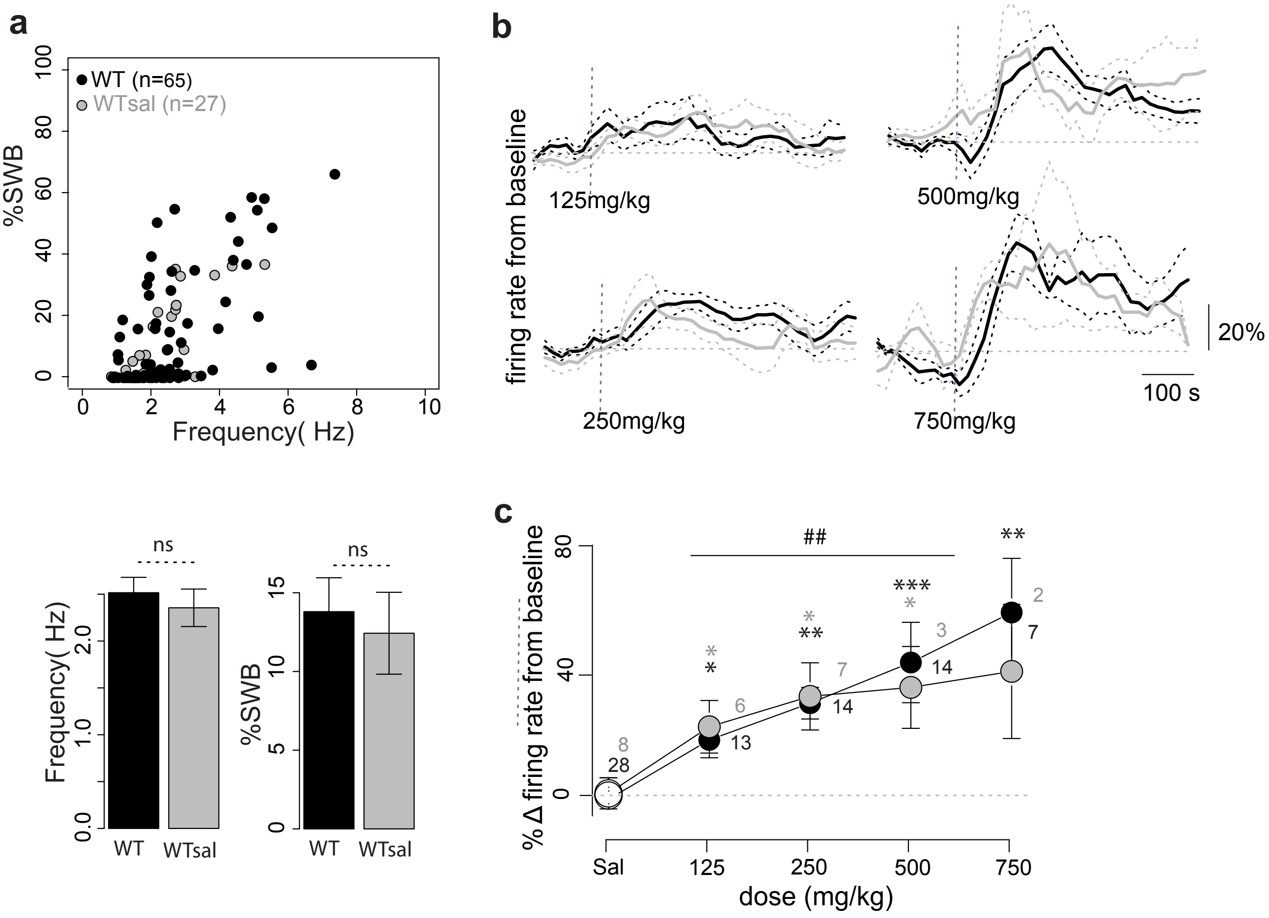

Supplementary Figure S2: Comparison between WT naïve mice and WTsaline mice

(a, top) Plot of mean frequency (Hz) against percentage of spikes within a burst (%SWB) of DA neurons in WT naïve (black, n=76) and WTsal mice (gray, n=27). (a, bottom) Barplot of the mean frequency (left) and % SWB (right) for the same groups. For frequencies: ns: $p>0.05$ t.test. For bursting activity: ns: $p>0.05$ Wilcoxon test. (b) Mean ± SEM DA cell firing frequency variations in WT naïve (black) and WTsal mice (gray) after injection of the indicated ethanol dose. Dotted vertical line indicates the time of the injection. (c) Dose-response curve for ethanol-elicited DA responses in WT naïve (black) and WTsal mice (gray). Mean ± SEM of the maximum of variation from baseline in firing frequency. Number of recorded neurons is indicated for each corresponding group. (Two-way ANOVA: dose effect: ##$p<0.01$, no group effect, nor group-dose interaction). Asterisks indicated significative effect of the tested dose (after vs before injections) ***$p<0.001$, **$p<0.01$, *$p <0.05$, Wilcoxon paired test.

Supplementary Figure 3

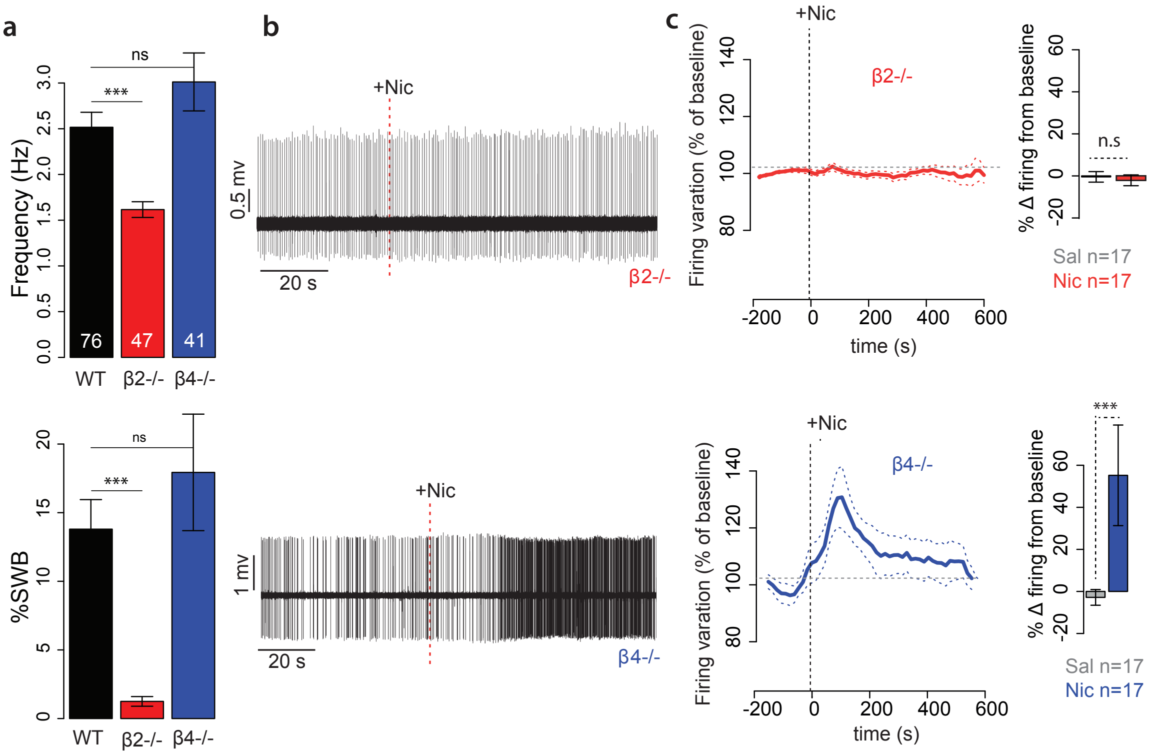

Supplementary Figure S3: Spontaneous activity and nicotine-induced responses in WT, β2-/- and β4-/- mice. (a) Barplot of the mean frequency (upper panel) and % SWB (lower panel) of DA neurons in WT (left, black, n=65), β2-/- (middle, red, n=47) and β4-/- mice (right, blue, n=41). For frequencies: ns: p>0.05; ***p<0.001, t-test with Bonferroni-holm's correction. For bursting activity: ns: p>0.05; ***p<0.001 Wilcoxon test with Bonferroni-holm's correction. (b) Typical electrophysiological recordings showing the responses to nicotine (30µg/kg) in β2-/- (upper panel) and β4-/- (lower panels). (c) Mean ± SEM DA cell firing frequency variation for β2-/- (upper panels) and β4-/- (lower panels) after nicotine injection compared with saline injection. Number of recorded neurons is indicated for each corresponding group. ***p<0.001, ns: p>0.05, Wilcoxon tests.

Supplementary Figure 4

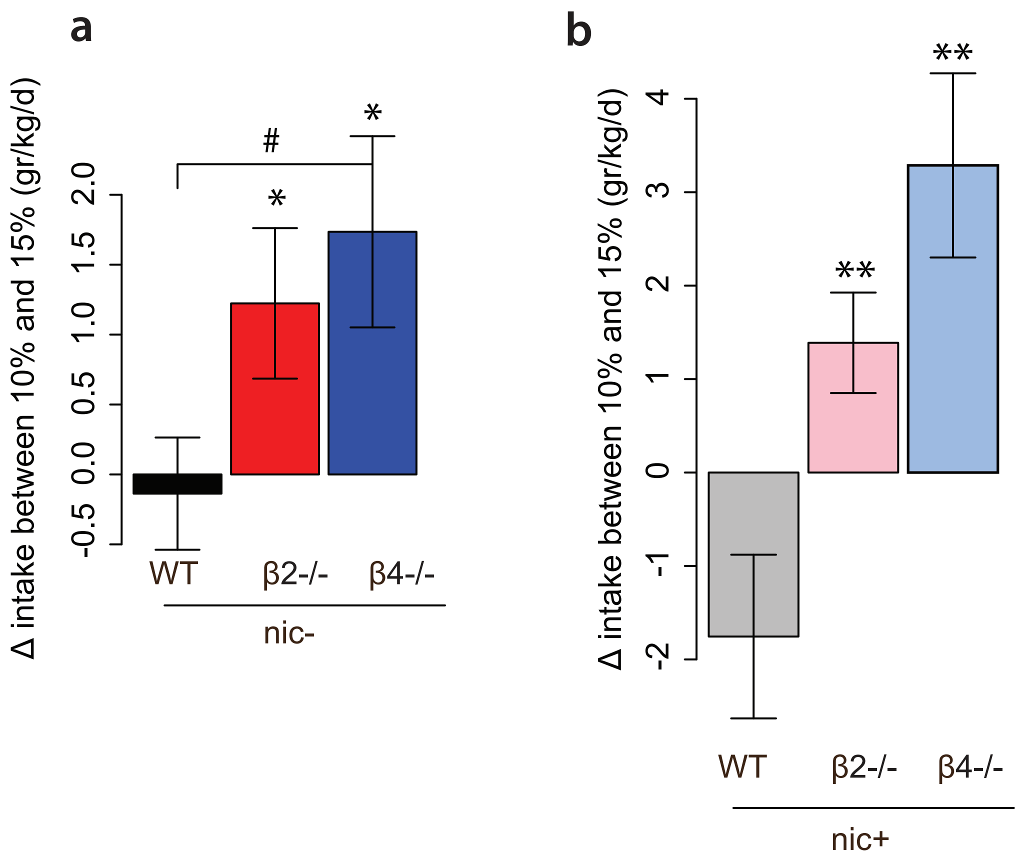

Supplementary Figure S4: Intake variation between the doses of 10% and 15%. (a) Mean ± SEM of alcohol intake variation when switching from the dose of 10% to the dose of 15% for WT (black, n=18), β2-/- (red, n=9) and β4-/- (blue, n=6) mice (Wilcoxon paired test: *$p < 0.05$; Wilcoxon test between groups: #$p<0.05$). (b) Mean ± SEM of alcohol intake variation when switching from the dose of 10% to the dose of 15% for WTnic+ (gray, n=15), β2nic+ (pink, n=8) and β4nic+ (lightblue, n=6) mice (Wilcoxon paired test: **$p < 0.01$).

Supplementary Figure 5

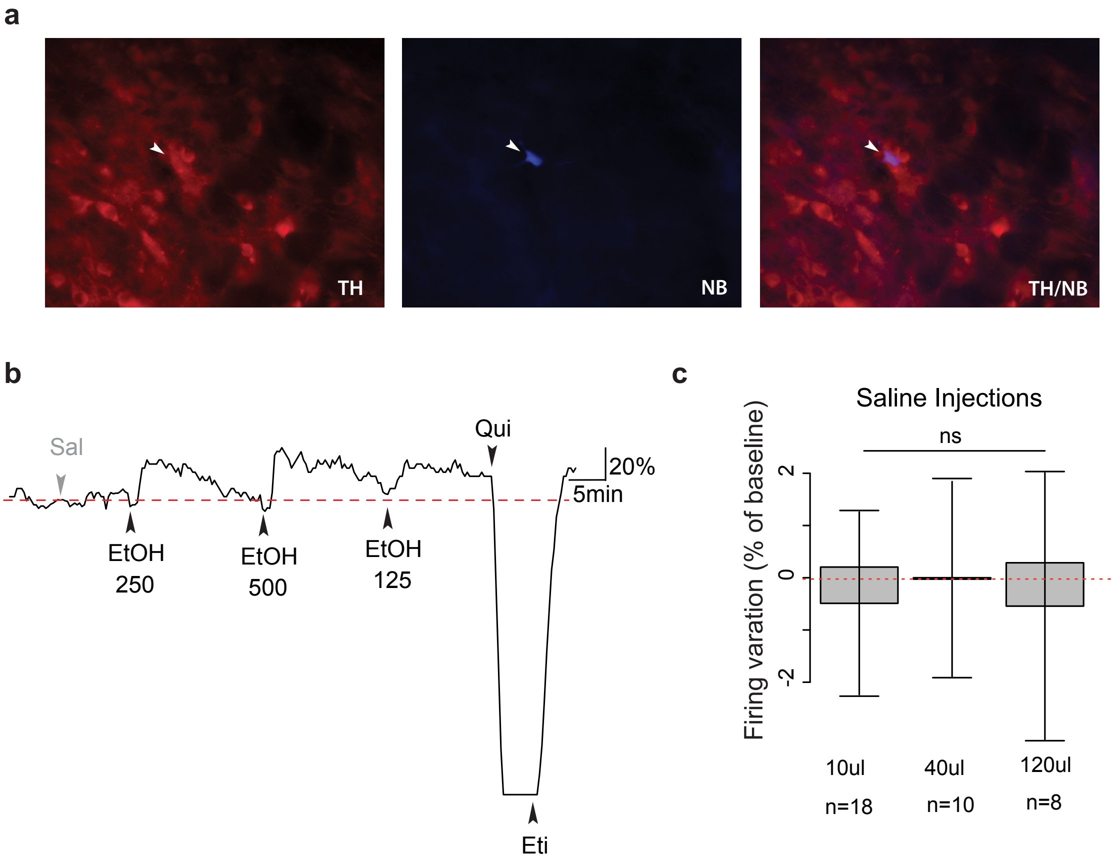

Supplementary Figure S5: Pharmacological and immunocytochemical identification of recorded neurons. (a) Representative photomicrographs of a DA neuron labelled with anti-TH (tyrosine-hydroxylase, left) and NB (Neurobiotine, middle) antibodies. TH and NB co-labelling on the right. White arrowheads indicate the recorded neurons. (b) Example responses of a VTA DA neuron subjected to pharmacological characterization. EtOH, Qui and Eti labelled arrowheads respectively represent the times of alcohol (125, 250 or 500 mg/kg), quinpirole (1 mg/kg), and eticlopride (1 mg/kg) intravenous injections. Red dotted lines represent baseline activity levels. (c) Barplot of the maximum of firing frequency variation from baseline (mean ± SEM) after an injection of 10 (left), 40 (middle) and 120µl (right) of saline. Number of recorded neurons is indicated for each corresponding group.
